# Supplementary material for: Longitudinally stable, brain‐based predictive models mediate the relationships between childhood cognition and socio‐demographic, psychological and genetic factors
Source: Hum Brain Mapp. 2022 Jul 28;43(18):5520–42. doi: 10.1002/hbm.26027 (PMC9704790; doi:10.1002/hbm.26027)
Supplement: Supplementary file 1 — Appendix S1 Supporting Information [file HBM-43-5520-s001.docx]

Supplementary Materials

Appendix 1: *g*-Factor Based on Different Confirmatory Factor Analysis Models

Here using the first-layer training data, we compared factor scores of the *g-*factor across three different confirmatory factor analysis (CFA) models: the 2^nd^-order model, the single-factor model, and the mixture between Exploratory Factor Analysis (EFA) and CFA models, here by referred to as the EFA-CFA model.

For the 2^nd^-order model (see Supplementary Figure 1), we had the *g*-factor as the 2^nd^-order latent variable. We also had three 1^st^-order latent variables in the model: language (underlying Picture Vocabulary and Oral Reading Recognition), mental flexibility (underlying Flanker and Pattern Comparison Processing), and memory recall (underlying Picture Sequence Memory and Rey-Auditory Verbal Learning). This 2^nd^-order model of the *g*-factor showed a good fit: (a) scaled, robust Comparative Fit Index (CFI) =.995, (b) scaled, robust Tucker-Lewis Index (TLI) =.988, (c) scaled, robust Root Mean Square Error of Approximation (RMSEA) = .029 (90%CI=.015-.043) and (d) robust Standardized Root Mean Square Residual (SRMR) = .014. The *g*-factor latent variable of the 2^nd^-order model also had high internal consistency: OmegaL2=.78. The 2^nd^-order model led to the sample-size adjusted Bayesian (BIC) at 46549.957.


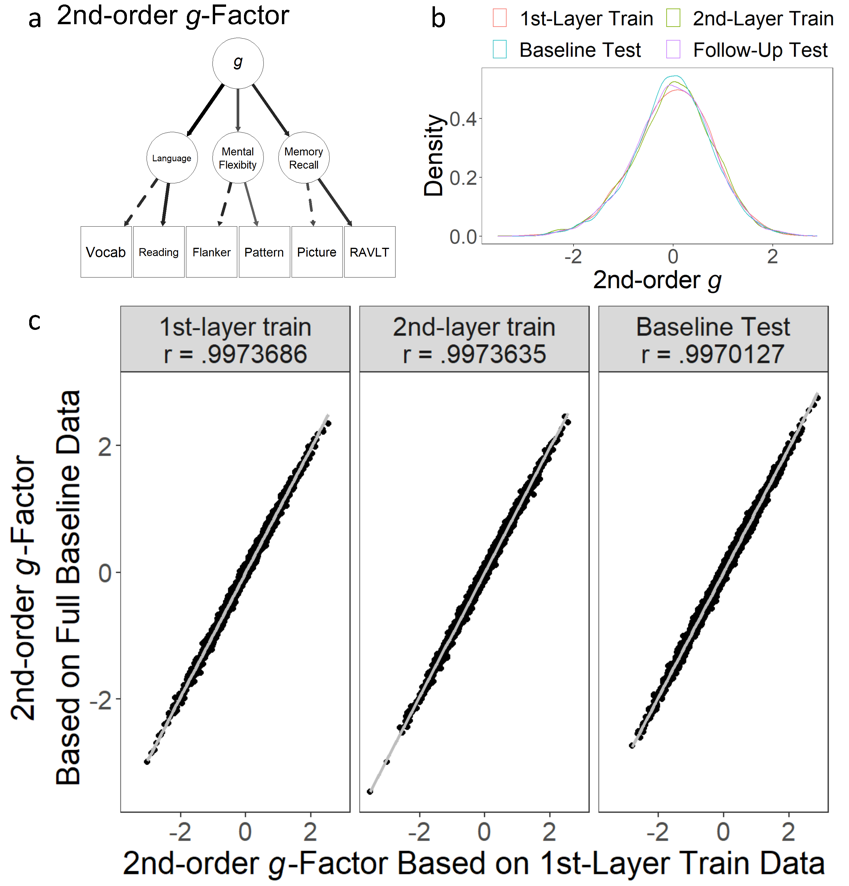


*Supplementary Figure 1. The 2^nd^-order model of the g-factor. 2A shows the 2^nd^-order model of the g-factor. Line thickness reflects the magnitude of standardized parameter estimates. The dotted lines indicate marker variables that were fixed to 1. Vocab = Picture Vocabulary; Reading = Oral Reading Recognition; Pattern = Pattern Comparison Processing; Picture = Picture Sequence Memory; RAVLT = Rey-Auditory Verbal Learning. 2B shows the distribution of the g-factor factor score across the four data splits. The distribution of the g-factor factor scores was similar across data splits.*

For the single-factor model (see Supplementary Figure 2), we had the *g*-factor as the only one latent variable, underlying variation in all manifest variables: Picture Vocabulary, Oral Reading Recognition, Flanker and Pattern Comparison Processing, Picture Sequence Memory and Rey-Auditory Verbal Learning. This single-factor model of the *g*-factor showed a numerically poorer fit: (a) scaled, robust Comparative Fit Index (CFI) =.828, (b) scaled, robust Tucker-Lewis Index (TLI) =.713, (c) scaled, robust Root Mean Square Error of Approximation (RMSEA) = .139 (90%CI=.129-.149) and (d) robust Standardized Root Mean Square Residual (SRMR) = .070. The single-factor model led to a poorer (i.e., higher) sample-size adjusted Bayesian (BIC) than the 2^nd^-order model, at 47028.981.


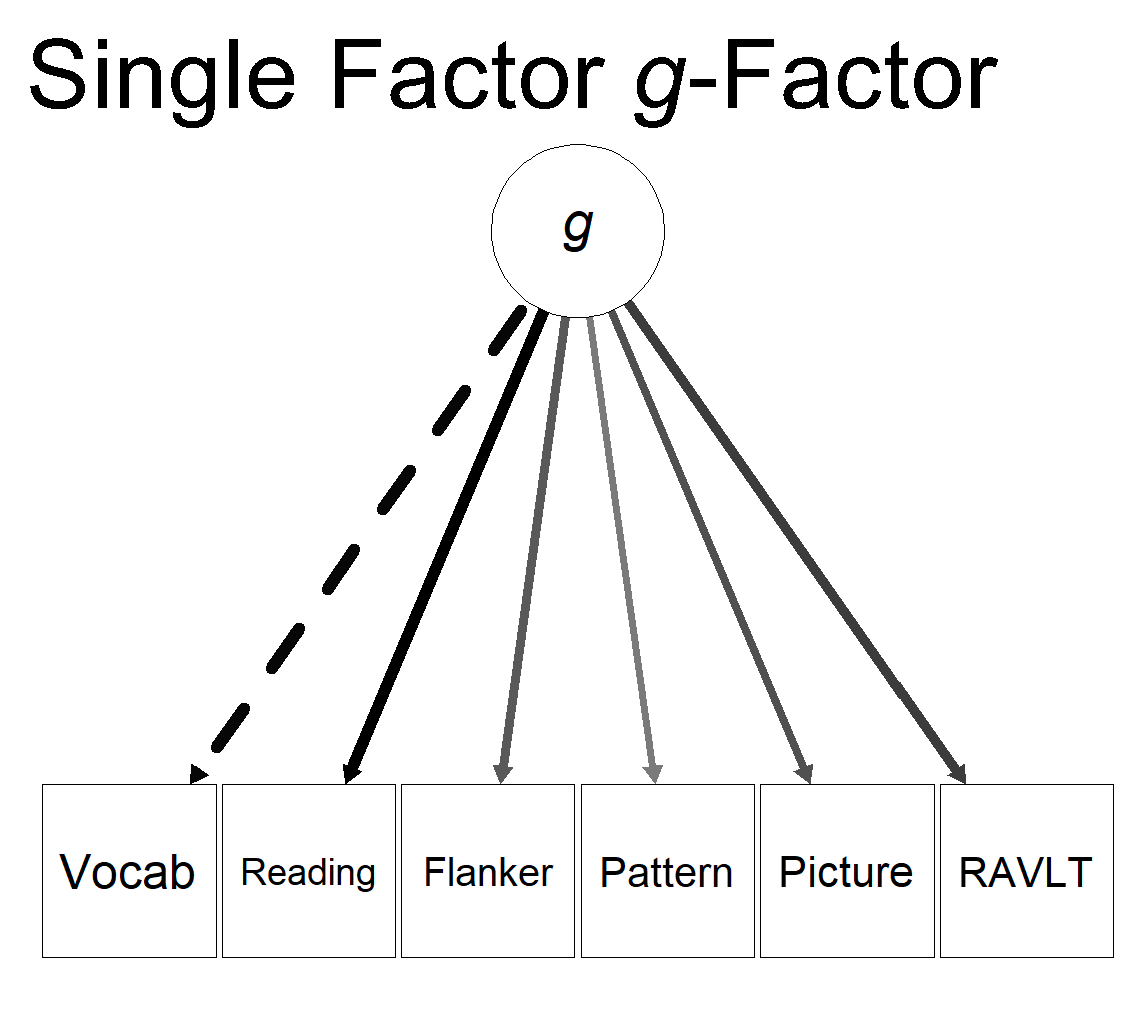


*Supplementary Figure 2. The single-factor model of the g-factor. Line thickness reflects the magnitude of standardized parameter estimates. The dotted lines indicate marker variables that were fixed to 1. Vocab = Picture Vocabulary; Reading = Oral Reading Recognition; Pattern = Pattern Comparison Processing; Picture = Picture Sequence Memory; RAVLT = Rey-Auditory Verbal Learning.*

For the EFA-CFA model (see Supplementary Figure 3), we first applied EFA to the performance across the six cognitive tasks via the psych package. To implement EFA, we first ran a parallel analysis to determine the number of factors to retain (see Supplementary Figure 3a). The parallel analysis suggested three as the number of factors. We then ran EFA with three factors using ‘oblimin’ as the rotation, ‘maximum likelihood’ as the factoring method and “Thurstone” as the factor scoring method. This resulted in the same three factors used in the 2^nd^-order CFA model (see Supplementary Figure 3b). See Supplementary Table 1 for the standardized loadings (pattern matrix) based upon the correlation matrix. We then extracted factor scores from the final EFA model and used them as the manifest variables in another CFA model (see Supplementary Figure 3c).

Perhaps due to the use of EFA factor scores as the manifest variables, the EFA-CFA model resulted in a model with the perfect fit (as reported by the lavaan package): (a) scaled, robust Comparative Fit Index (CFI) = 1, (b) scaled, robust Tucker-Lewis Index (TLI) = 1, (c) scaled, robust Root Mean Square Error of Approximation (RMSEA) = 0 (90%CI= 0-0) and (d) robust Standardized Root Mean Square Residual (SRMR) = 0. The sample-size adjusted Bayesian (BIC) of the EFA-CFA was the best (i.e., lowest) among the three models at 17197.366.


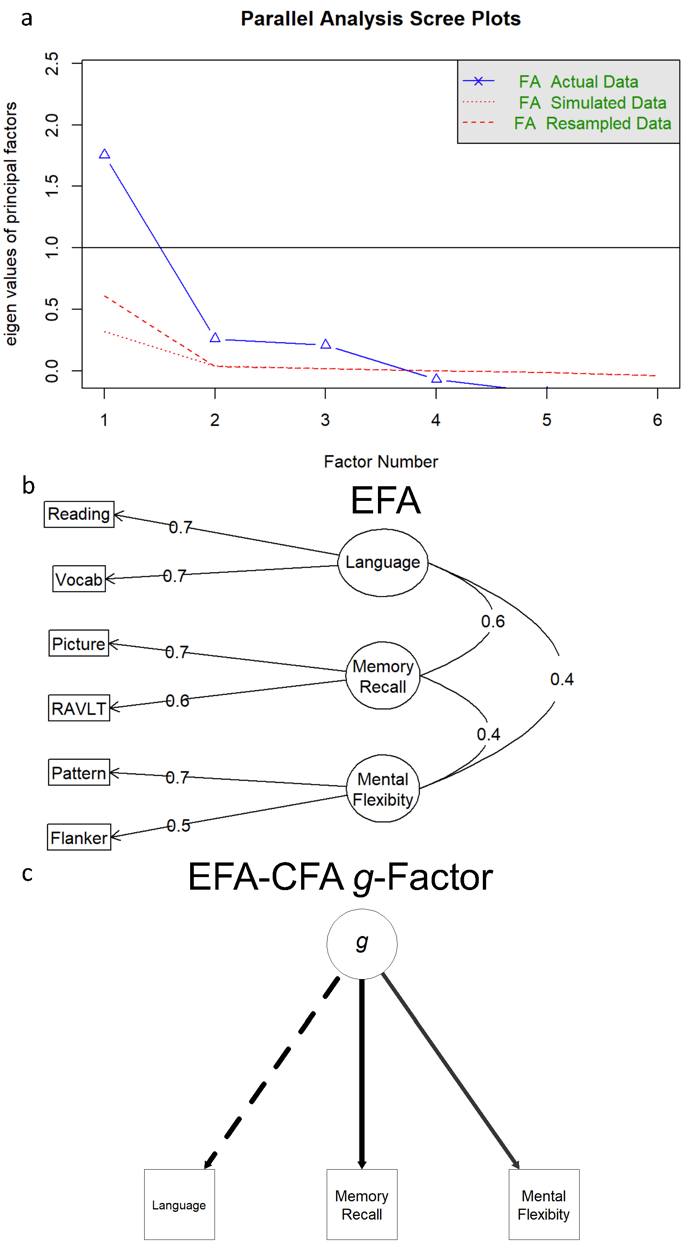


*Supplementary Figure 3. The EFA-CFA model of the g-factor. 3A shows the parallel analysis scree plot, suggesting three factors as the most likely solution for the EFA model. 3B shows the three-factor EFA model. 3C shows the EFA-CFA model, where its manifest variables were factor scores of the EFA model. Line thickness reflects the magnitude of standardized parameter estimates. The dotted lines indicate marker variables that were fixed to 1. Vocab = Picture Vocabulary; Reading = Oral Reading Recognition; Pattern = Pattern Comparison Processing; Picture = Picture Sequence Memory; RAVLT = Rey-Auditory Verbal Learning.*

Supplementary Table 1. Standardized loadings based upon correlation matrix

|  | Language | Memory Recall | Mental Flexibility | h2 | u2 | com |
| --- | --- | --- | --- | --- | --- | --- |
| Picture Vocabulary | **0.7** | 0.01 | 0.01 | 0.51 | 0.49 | 1 |
| Oral Reading Recognition | **0.73** | 0.01 | 0.01 | 0.55 | 0.45 | 1 |
| Flanker | 0.15 | -0.01 | **0.53** | 0.37 | 0.63 | 1.2 |
| Pattern Comparison | -0.05 | 0.02 | **0.68** | 0.44 | 0.56 | 1 |
| Picture Sequence Memory | -0.06 | **0.69** | 0.04 | 0.45 | 0.55 | 1 |
| RAVLT | 0.13 | **0.58** | -0.04 | 0.41 | 0.59 | 1.1 |

We then examined the similarity in the factor scores of the *g*-factor based on three different CFA models at different data splits. We first extracted factor scores from the data used for fitting the CFA models, the 1^st^-layer training set, as well as unseen data sets, including the 2^nd^-layer training data, baseline test set and follow-up test data. Across data sets, we found high similarity in the factor scores of the *g*-factor across the three different CFA models at Pearson’s *rs* ≥ .987 (see Supplementary Figure 4). Accordingly, the choice of *g*-factor models had only minimal effects on the estimation of the factor scores for the *g*-factor.


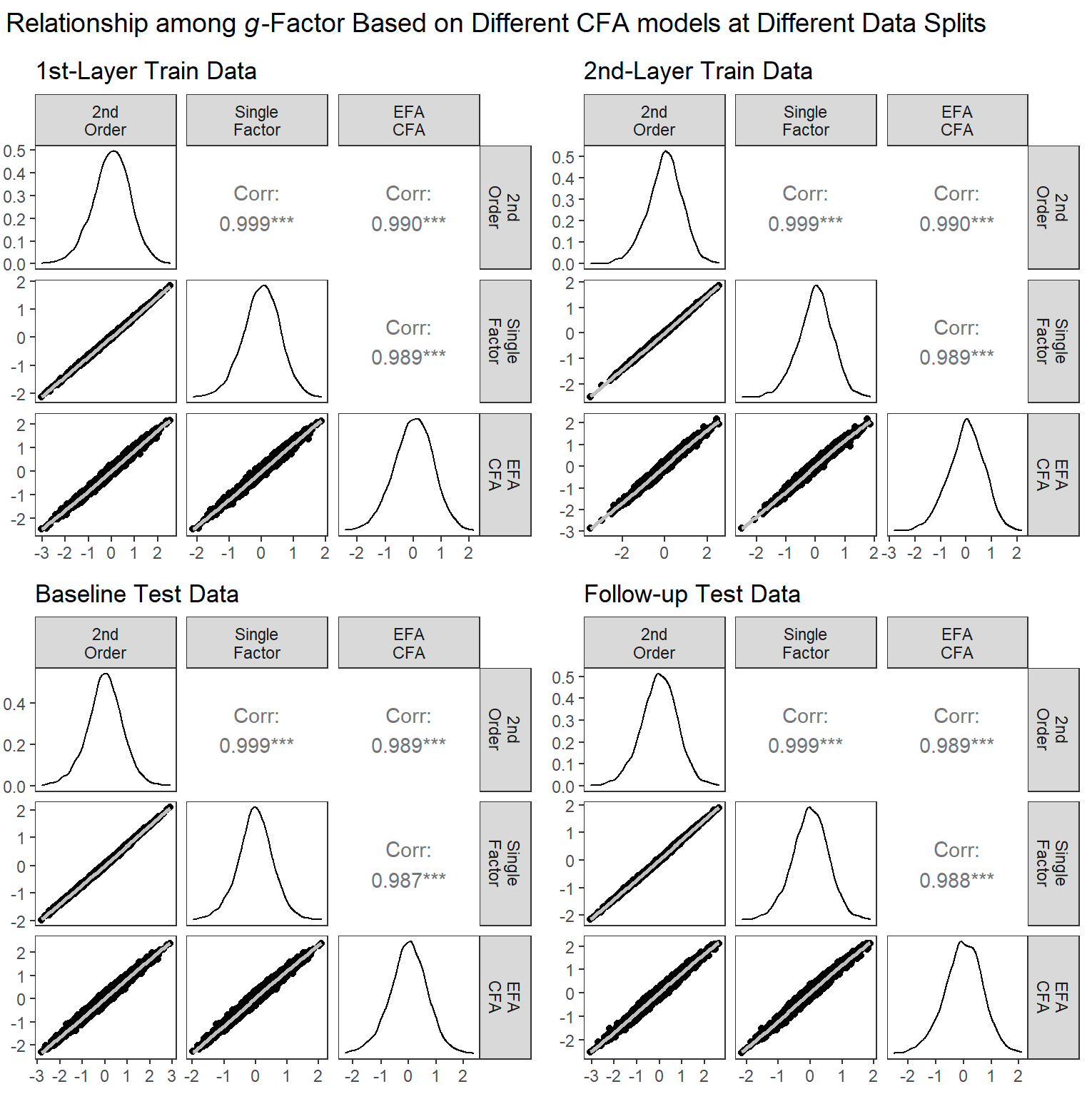


*Supplementary Figure 4. The Relationship in the factor scores of the g-factor based on three different CFA models at different data splits.*

Appendix 2: 2^nd^-order g-Factor based on different sets of data

To demonstrate the stability of the factor scores of the *g*-factor when applied to unseen data (i.e., not part of the modelling process), we compared the *g*-factor scores estimated from the first-layer training data vs. the scores estimated from the full baseline data. Using the first-layer training data to build the 2^nd^-order model led to highly similar factor scores of the *g*-factor to using the full baseline data across different data splits, at Pearson’s *rs* > .997 (see Supplementary Figure 5). Note we did not use the follow-up test data here since the follow-up test data included the same participants as the baseline test data, and including the data from the same participants twice may lead to a biased model.


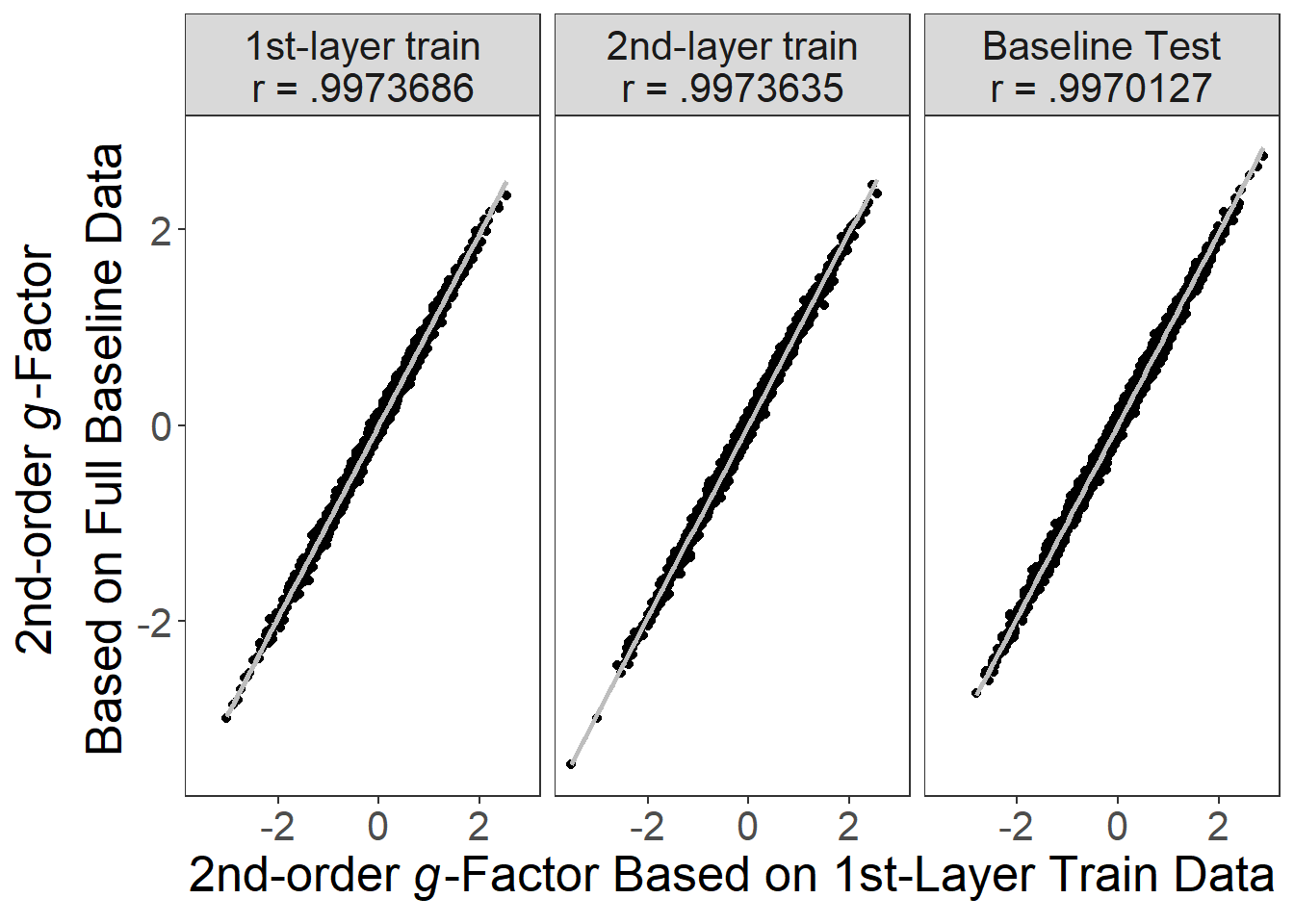


*Supplementary Figure 5. The similarity in* the factor sco*res of the g-factor of the 2^nd^-order model based on whether the model was built from the 1^st^-layer training data or the full baseline data.*

Appendix 3: Hyperparameters for the Longitudinal Predictive Models for Multimodal MRI

From the first-layer training set, we found the best-tuned Elastic Net’s mixture was 0 for N-Back task-based fMRI, rs-fMRI and DIT, .1 for sMRI, and 1 for SST and MID task-based fMRI. Thus, whether to have brain features shrunk together (the Ridge solution, mixture close to 0) or to have certain features from all features selected (the Lasso solution, mixture close to 1) depended on the modality. The averaged Elastic Net’s penalty was .339 (SD = .45). From the second-layer training set, we found the best-tuned Random Forests’ mtry (the number of features randomly sampled at each path split) at 4 and min_n (the minimum number of observations in a node per path split) at 170.
